# Supplementary material for: Risk Factors Associated with Dengue Virus Infection in Guangdong Province: A Community-Based Case-Control Study
Source: Int J Environ Res Public Health. 2019 Feb 20;16(4):617. doi: 10.3390/ijerph16040617 (PMC6406885; doi:10.3390/ijerph16040617)
Supplement: Supplementary file 1 [file ijerph-16-00617-s001.pdf]

A. Basic information

Z1. Investigation unit: ______________________

Z2. Name: __________________________

Z3. Telephone number: __________________________

Z4. Questionnaire number: ________

A1. Residence

1. Permanent residents 2. Floating population (migrants who live locally for 6 months or less)

A2. The number of household residents

1. 1 person 2. 2-3 persons 3. 4-5 persons 4. ≥6 persons

A3. Per capita family income monthly (￥)

1. <2000 2.2000-5000 3. 5000-8000 4. ≥8000

A4. Blood type

1. A 2. B 3. O 4. AB 5. Unknown

The following questions are answered according to the current situation of blood extraction:

A5. Have you ever suffered from dengue fever in the past?

1. Yes (A5.1 Data <yyyy/mm/dd> such as:2015-01-01) 2. No

A6. Have you ever lived/worked with patients with dengue fever in the past?

1. Yes (A6.1 the number of patients__________) 2. No

A7. Do you have outbound tourism experience? (If no, please answer A9)

1. Yes 2. No

A8. If yes, which country have you been to?

A8.1 Country______________________, A8.2 Data_____year A8.3___month

A8.4 Country _____________________, A8.5 Data_____year A8.6___month

A9. Do you have activities in the park?

1.Yes 2. No

A10.Do you have outdoor sports (Hiking, camping, etc.)

1.Yes 2. No

A11. The frequency of domestic sewage disposal:

1. Everyday 2. Two days 3. Three days and above

4. No domestic sewage

A12. The frequency of garbage disposal:

1. Everyday 2. Two days 3. Three days and above

A13. Do you participate in the community hygiene management intervention?

1.Yes 2. No

B. Environmental factors

B1. The geographical location of the current residence:

1. Rural 2. City 3. Urban-rural integration

B2. The structure of housing building structure:

1. Brick-wood structure 2. Brick-wood and concrete 3. Concrete structure

B3. Housing type:

1. Single-family apartment   2. Commercial residential community   3. Villa

B4. The age of the housing (year):

1. <10   2. 10-20   3. 20-40   4. >40

B5. The floor number of current housing:

1. 1-3   2. 4-9   3. ≥10

B6. Average numbers of person per room:

1. 1   2. 2   3. ≥3

B7. The area of housing (m2):

1. <50   2. 51-100   3. 101-150   4. >150

B8. Do you use air-condition use in current residence?

1.Yes   2. No

B9. How about the quality of indoor daylight in your current residence?

1.Yes   2. No

B10. How about the effect of Ventilation in your current residence?

1.Yes   2. No

B11. Do you keep pets in your current residence?

1.Yes   2. No

B12. Do you raise poultry in your current residence?

1.Yes   2. No

B13. Do you breed aquatic plants in your current residence?

1.Yes   2. No

B14. Do you use mosquito nets in your current residence?

1.Yes   2. No

B15. Do you use mosquito repellent in your current residence?

1.Never   2. Occasionally   3. Often

B16. Do you use electronic mosquito killing facilities in your current residence?

1. Never   2. Occasionally   3. Often

B17. Do you use camphor in your current residence?

1. Never   2. Occasionally   3. Often

B18. Are there garbage collection sites within 200m around housing?

1. Yes   2. No

B19. Are there junk yards within 200m around housing?

1. Yes   2. No

B18. Are there ponds within 200m around housing?

1. Yes   2. No

B18. Are there construction sites within 200m around housing?

1. Yes   2. No
